# Improved YOLOv10: A Real-Time Object Detection Approach in Complex Environments

**DOI:** 10.3390/s25226893

**Published:** 2025-11-12

**Authors:** Qili Wu, Xin Nie

**Affiliations:** School of Computer Science and Engineering, Wuhan Institute of Technology, Wuhan 430079, China; 22307010053@stu.wit.edu.cn

**Keywords:** YOLOv10, BiFPN, Squeeze-and-Excitation, small target detection, occlusion-robust detection

## Abstract

Object detection of small and occluded targets in complex scenarios is a vital yet challenging task in computer vision, with applications in intelligent systems (e.g., kitchen safety supervision). To address limitations of existing models, this study proposes an improved YOLOv10 algorithm with three key innovations. We first introduce a Mosaic-9 data augmentation strategy to enhance small target density in training samples. The traditional PANet in YOLOv10 is replaced by the Bidirectional Feature Pyramid Network (BiFPN), which uses cross-scale bidirectional connections and learnable weights to optimize multi-scale feature fusion. A Squeeze-and-Excitation (SE) channel attention module is integrated into the CSPDarknet backbone to emphasize key feature channels and mitigate background interference for occluded objects. Experiments on a self-constructed dataset (6508 images with multi-scale and occluded targets) show the improved YOLOv10 achieves 69.5% mAP@0.5, a 7.7 percentage point increase over YOLOv10n, while maintaining 12.1 ms inference speed. Ablation studies verify Mosaic-9 enhances small target perception, BiFPN boosts mAP@0.5 by 5.7%, and SE improves occlusion robustness by 4.8%. This work offers a generalizable multi-module optimization framework for YOLO-series models, applicable to various small and occluded target detection tasks, advancing lightweight object detection algorithms and intelligent vision systems.

## 1. Introduction

In the modern food processing and catering service industry, a standardized dress code for employees (including wearing chef hats, masks, and work clothes) is a basic requirement to ensure food safety and hygiene. As one of the core technologies of intelligent kitchen supervision systems, object detection can provide monitoring information on personnel behavior norms for catering kitchens, effectively ensuring food safety. However, in practical kitchen scenes, diverse personnel postures, frequent occlusions, and complex backgrounds interfere with the feature extraction of hats and masks. For small and partially occluded targets, effective feature extraction becomes difficult, reducing detection accuracy and compromising food safety supervision. In this context, traditional object detection methods and deep learning-based object detection methods are used to identify and locate objects of interest in images or videos [1]. Among them, traditional object detection methods [2,3] have simple calculations and good interpretability, and are suitable for object detection tasks in simple scenarios. However, they perform poorly in complex environments, have slow detection speeds, and rely on manual design for feature extraction. In contrast, deep learning-based object detection methods [4,5] have been widely used due to their powerful feature learning ability, robustness, and adaptability, becoming the mainstream method in the field of modern object detection [6].

Currently, deep learning-based object detection methods mainly include two-stage detection algorithms and single-stage detection algorithms. Among them, two-stage detection algorithms, represented by the R-CNN series [7,8], have high detection accuracy but slow training and detection speed, making it difficult to deploy and apply. In contrast, single-stage detection algorithms such as YOLO series [9,10,11,12,13,14] have significantly improved detection speed while meeting the required detection accuracy, and are widely used in object detection tasks. With the continuous development of object detection technology, researchers are gradually shifting their focus to key challenges in practical scenarios, such as small object detection, recognition of occluded targets, and feature extraction in complex backgrounds. Solving these problems will further enhance the accuracy, robustness, and adaptability of object detection algorithms in different scenarios.

Due to the insufficient feature expression ability of hats and masks, as well as the inadequate processing ability of occluded targets, existing algorithms have poor performance in object detection in kitchen scenes. To further improve the detection performance of the algorithm, researchers have proposed various improvement strategies. Liu Yi et al. [15] introduced the BottleneckCSP module in YOLOv5s and added a large-scale feature fusion structure. At the same time, they integrated SE attention mechanism into the network structure, making the network’s autonomous learning more focused on small target feature channels, effectively improving the detection ability of small targets. Jiang Xinling et al. [16] improved the model structure of the YOLOX_s algorithm by establishing a new predictive feature layer that effectively integrates high-level semantic information and low-level positional information to predict small targets. They changed the BCE-Loss of obj_loss to Focal_Loss and used Focal_Loss to train the obj branch to reduce missed detections and enhance the detection ability of occluded targets. Pei Yunxia et al. [17] used depthwise separable convolution to replace some convolutions in the YOLOv4 model, and replaced the feature pyramid with a recursive feature pyramid to improve the feature learning ability of small and occluded targets. Afterwards, the K-means++ algorithm was introduced to adaptively obtain anchor boxes, optimize the initial values of anchor boxes, and use the complete intersection to union ratio and cross entropy to construct a loss function. The improved YOLOv4 was trained and the enhanced image was input to achieve occlusion target recognition. Cao Jiwei et al. [18] improved the detection performance of the model for small targets by reconstructing the backbone network, introducing attention mechanisms, and optimizing the loss function. However, the detection performance of occluded targets has not been effectively improved. Wu Shaobin et al. [19] designed a multi-frame feature fusion module that propagates historical frame features to the current frame for fusion, improving the algorithm’s detection accuracy for occluded targets. However, this method has limitations in detecting small targets. Yang Lei et al. [20] enhanced the detection ability of the model for small and occluded targets by constructing a feature receptive field fusion module and adding a shallower feature layer P2 detection head. However, the model has a large number of parameters and the detection speed struggles to meet real-time requirements.

In order to address the issues of insufficient detection accuracy for small and occluded targets in clothing detection within kitchen environments, as well as the limited adaptability of traditional methods to dynamic scenes, which hampers their ability to meet real-time requirements, this paper proposes an improved YOLOv10n-based detection algorithm for hats and masks in kitchen scenes. To start with, the Mosaic-9 data augmentation strategy is employed to enhance the model’s sensitivity to small targets by generating training samples containing densely distributed small objects. To address intermediate feature loss, the PANet module in YOLOv10n is replaced with a BiFPN fusion module to improve the model’s detection performance for small targets. For occluded object detection, an attention mechanism is introduced to enhance the focus on key target regions (e.g., head and face areas). Specifically, within the backbone network, the channel attention SE module is integrated into the residual blocks of CSPDarknet to enhance the model’s capability in detecting occluded targets. Experimental results demonstrate that the improved model achieves significant improvements in both detection accuracy and real-time performance compared to the baseline YOLOv10n model. These enhancements ensure high detection accuracy while maintaining real-time efficiency, thereby providing a reliable technical solution for automated clothing detection in kitchen environments.

## 2. Principle of YOLOv10

**Figure 1:**
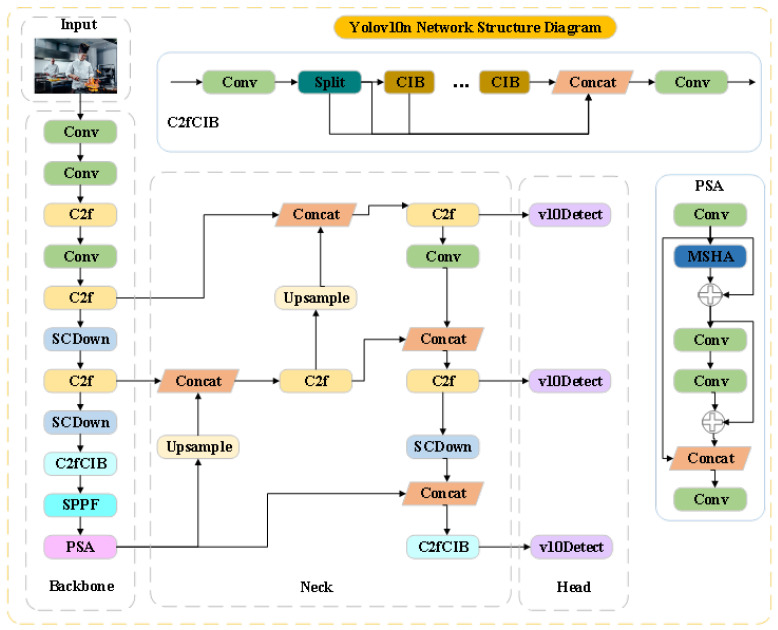
YOLOv10n Network Structure Diagram. Illustration of the YOLOv10n architecture, including Backbone, Neck, and Head modules, showing the flow of feature extraction and object detection.

As the latest version of the YOLO series object detection algorithm, YOLOv10 has made significant progress in model architecture and performance. The core architecture of this algorithm consists of three key components: Backbone, Neck, and Head, as shown in Figure 1.

Among YOLOv10 variants (nano/s/m), we select YOLOv10n as the baseline primarily for three reasons: first, its parameter size (2.7 M) is smaller than that of YOLOv10s (4.5 M) and YOLOv10m (8.8 M), which supports lightweight deployment on edge devices in kitchen supervision systems; second, its architectural compatibility with the proposed BiFPN and SE modules—its simplified CSPDarknet backbone avoids redundant feature conflicts when integrating attention mechanisms; third, compared with YOLOv8n (3.0 M parameters), YOLOv10n’s dynamic label allocation strategy better adapts to the uneven distribution of small/occluded targets in kitchen scenes, laying a foundation for subsequent performance improvements.

Compared to previous versions, the YOLOv10n backbone network adopts an improved CSPNet (Cross Stage Partial Network) structure [21], which significantly reduces computational complexity while maintaining high feature extraction capabilities through carefully designed cross stage local connections and channel attention mechanisms. Specifically, the network consists of multiple CSP modules, each consisting of a basic convolutional layer, batch normalization layer, and LeakyReLU activation function, and introduces residual connections to alleviate the gradient vanishing problem. The neck network adopts the Path Aggregation Network (PANet) structure [22], which achieves deep fusion of feature maps of different scales through bidirectional feature pyramids from top to bottom and from bottom to top [23]. This structure can effectively combine deep semantic information with shallow detail features, which is beneficial for detecting targets of different sizes. However, the standard PANet suffers from information loss during the feature fusion process, especially in terms of insufficient feature preservation for small targets, which directly affects the detection performance of kitchen dressing small targets. The detection head adopts a Decoupled Head design, which separates the classification task and regression task for processing. This design effectively reduces interference between two tasks and improves detection accuracy. The classification branch uses convolutional layers with sigmoid activation functions to predict the class probability of each anchor box, while the regression branch predicts the coordinate offset of bounding boxes. In addition, YOLOv10 also introduces a dynamic label allocation strategy that dynamically adjusts the allocation of positive and negative samples based on prediction quality, further improving training efficiency.

Despite the advantages of YOLOv10n, its PANet neck still has two limitations in kitchen scenes: first, equal-weight feature fusion fails to prioritize small target features (e.g., masks), leading to information loss; second, the lack of attention mechanisms in CSPDarknet makes it difficult to suppress background interference (e.g., kitchen utensils) for occluded targets. These limitations motivate the subsequent improvements—replacing PANet with BiFPN to optimize feature fusion, and integrating SE modules to enhance occlusion robustness.

## 3. Improved YOLOv10 Object Detection Algorithm

### 3.1. Improved Model Network Structure

In order to solve the problem of insufficient accuracy in detecting small and occluded objects in current kitchen clothing detection, and to improve the recognition performance of the model under various complex environmental influences, this paper proposes an improved YOLOv10 object detection algorithm. The improved model first preprocesses the input image through the Mosaic-9 data augmentation module, and then adds the BiFPN fusion module and attention mechanism module, respectively, to enhance the detection effect of the algorithm on small targets and occluded targets. These improvements work together to enhance the detection performance of the model in complex kitchen environments. The improved model network structure is shown in Table 1.

### 3.2. Mosaic Enhanced Network

**Figure 2:**
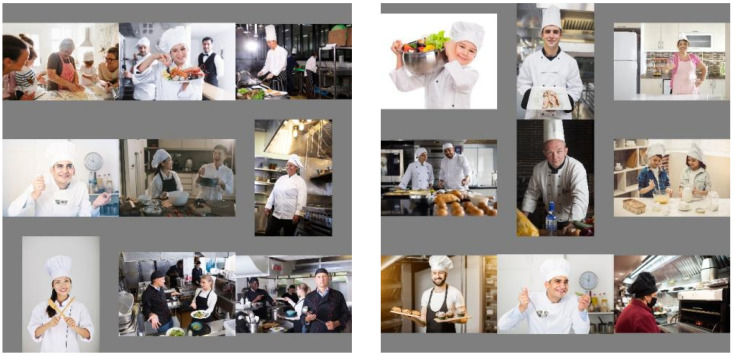
Mosaic-9 Data Augmentation Example. Demonstration of generating dense small-target samples via Mosaic-9 strategy.

Although traditional data augmentation methods such as Mosaic 4 can increase the diversity of training samples, their effectiveness in enhancing small targets is limited. Our proposed Mosaic-9 [24] enhancement strategy significantly improves the detection performance of small targets through the following innovative designs:

By randomly selecting 9 original training images and adjusting them to a uniform size (640 × 640), gray padding (127) was used instead of traditional zero padding to stitch the image blocks into a 1920 × 1920 composite canvas in a 3 × 3 grid. During the stitching process, adaptive mapping of bounding box coordinates was achieved by calculating the scaling ratio and grid offset of each subgraph, ensuring strict alignment between the stitched annotation information and the image. In response to the background interference problem of traditional Mosaic methods, an innovative fixed grayscale filling strategy is introduced to effectively avoid abrupt boundaries between the filling area and the image content. At the same time, the video memory usage is reduced by compressing the output size to 640 × 640, allowing the batch size to be flexibly set to 9. It is worth noting that this strategy naturally increases the appearance density of small targets in the training samples through multi-image stitching, especially suitable for dense scenes of small-sized targets such as chef hats and masks. The number of targets in the composite image is more than three times higher than the average single image. Through the 9-figure random sampling mechanism, the exposure probability of low-frequency categories is implicitly increased, thereby achieving a more balanced category distribution with limited training data. The partial samples of the enhanced Mosaic 9 dataset are shown in Figure 2.

To indirectly verify Mosaic-9’s effectiveness, we compared the dataset before and after augmentation. The average number of small targets per image increased from 1.2 to 3.8, and occluded targets increased from 0.8 to 2.3. This directly proves Mosaic-9’s role in enriching small/occluded target samples, which underpins the improved model’s performance on these targets.

### 3.3. BiFPN Fusion Module

**Figure 3:**
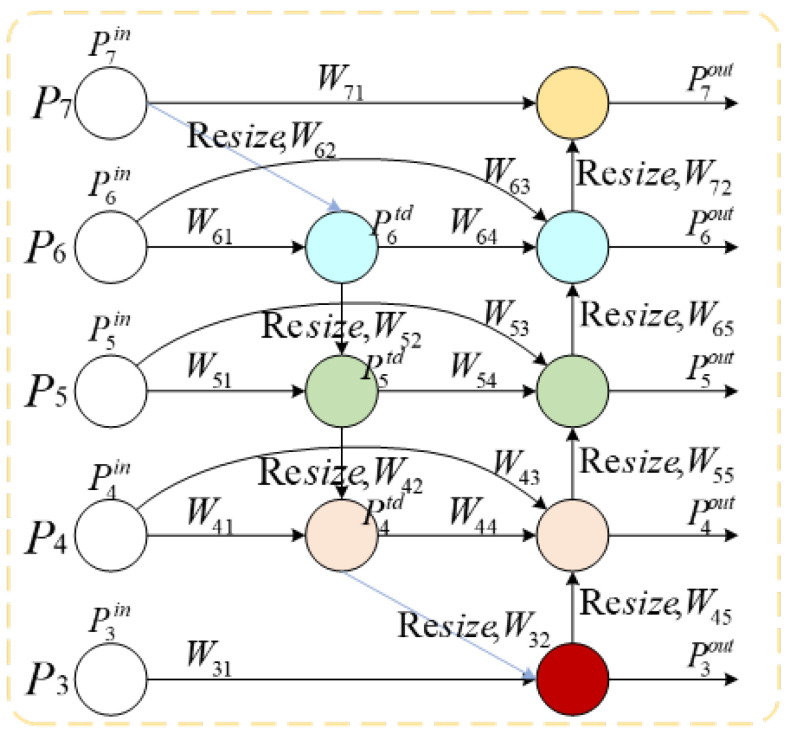
BiFPN module. Diagram of the BiFPN (Bi-directional Feature Pyramid Network) module, illustrating multi-scale feature fusion with weighted connections and resize operations.

The neck network of YOLOv10n employs the PANet module, a feature pyramid network structure originally proposed by Liu et al. [25], which has been widely adopted in object detection tasks. However, the standard PANet applies a uniform processing strategy to all input features, without differentiating the importance of features at various levels for specific tasks. Consequently, features of small targets may be overwhelmed by stronger semantic features during the fusion process, leading to insufficient preservation of small target features and negatively impacting the detection performance of small objects in kitchen clothing. To address the limitations of the original PANet in feature fusion, this paper introduces a BiFPN fusion module that dynamically adjusts the contribution weights of different input features through learnable parameters. This enables the model to autonomously learn and enhance the representation of critical features—such as detailed features of small targets—during training, while suppressing interference from redundant information [26]. The structure of the BiFPN module is illustrated in Figure 3.

For each input feature branch, the BiFPN fusion module initializes a learnable parameter $w_{i}$, which the dimension is consistent with the number of input features, and ensures that the weight is non negative through the ReLU activation function to avoid the feature offset problem caused by negative weight. The calculation formula is

(1) $$w_{i}^{\prime }=ReLU\left(w_{i}\right).$$

In order to ensure the interpretability and stability of the weight, it is also necessary to normalize the activated weight. The calculation formula is

(2) $${\hat{w}}_{i}=\frac{w_{i}^{\prime }}{\sum_{j=1}^{n}w_{j}^{\prime }+\epsilon },$$

where $\epsilon =10^{-4}$ is a tiny constant to prevent the denominator from being zero, *n* is the number of input features, and the sum of the normalized weights ${\hat{w}}_{i}$ is 1, which can directly characterize the relative importance of each input feature. Finally, the normalized weights are multiplied with the corresponding input features, and the final fused features are obtained by element-wise summation,

(3) $$Out=\sum_{i=1}^{n}{\hat{w}}_{i}\;\cdot \;x_{i},$$

where $x_{i}$ is the i-th input feature and Out is the fused output feature.

The BiFPN fusion module removes redundant connections that contribute less to feature fusion by analyzing the contribution of each node to the final detection result, reducing computational complexity while maintaining the performance of feature fusion. The BiFPN fusion module also adds an additional input path for input and output nodes at the same level to enhance feature fusion without increasing too much cost. At the same time, each bidirectional path is treated as a feature network layer and repeated multiple times to achieve higher-level feature fusion. The weighted calculation formula for the features of each output layer is as follows:

(4) $$P_{7}^{\mathrm{out}}=\mathrm{Conv}\left(\frac{P_{7}^{\mathrm{in}}\;\cdot \;W_{71}+\mathrm{Resize}\left(P_{6}^{\mathrm{out}}\right)\;\cdot \;W_{72}}{W_{71}+W_{72}+\epsilon }\right)$$

(5) $$P_{6}^{\mathrm{out}}=\mathrm{Conv}\left(\frac{P_{6}^{\mathrm{in}}\;\cdot \;W_{63}+P_{6}^{\mathrm{td}}\;\cdot \;W_{64}+\mathrm{Resize}\left(P_{5}^{\mathrm{out}}\right)\;\cdot \;W_{65}}{W_{63}+W_{64}+W_{65}+\epsilon }\right)$$

(6) $$P_{5}^{\mathrm{out}}=\mathrm{Conv}\left(\frac{P_{5}^{\mathrm{in}}\;\cdot \;W_{53}+P_{5}^{\mathrm{td}}\;\cdot \;W_{54}+\mathrm{Resize}\left(P_{4}^{\mathrm{out}}\right)\;\cdot \;W_{55}}{W_{53}+W_{54}+W_{55}+\epsilon }\right)$$

(7) $$P_{4}^{\mathrm{out}}=\mathrm{Conv}\left(\frac{P_{4}^{\mathrm{in}}\;\cdot \;W_{43}+P_{4}^{\mathrm{td}}\;\cdot \;W_{44}+\mathrm{Resize}\left(P_{3}^{\mathrm{out}}\right)\;\cdot \;W_{45}}{W_{43}+W_{44}+W_{45}+\epsilon }\right)$$

(8) $$P_{3}^{\mathrm{out}}=\mathrm{Conv}\left(\frac{P_{3}^{\mathrm{in}}\;\cdot \;W_{31}+\mathrm{Resize}\left(P_{4}^{\mathrm{td}}\right)\;\cdot \;W_{32}}{W_{31}+W_{32}+\epsilon }\right)$$

Among them, $P_{i}^{\mathrm{out}}$ represents the feature output of the i-th layer, Conv represents the convolution operation of feature processing, $P_{i}^{\mathrm{in}}$ represents the feature input of the i-th layer, $W_{ij}$ represents the learnable fusion weight, $P_{i}^{\mathrm{td}}$ represents the intermediate features of the i-th layer in the top-down path, and Resize represents the upsampling or downsampling in the resolution matching process, $\epsilon =10^{-4}$.

Compared with the equalization fusion strategy of PANet, the BiFPN fusion module introduced in this paper has many advantages. Its dynamic adaptability is reflected in the fact that through end-to-end training, the weight parameters can automatically adapt to the target distribution characteristics in the kitchen scene, and assign higher weights to small target features to enhance their proportion in the fusion results. The lightweight design makes the module only add *n* learnable parameters (*n* is the number of input features) and almost does not increase the computational overhead, which is suitable for deployment in resource-constrained edge devices. At the same time, the module does not need to manually design fusion rules and can adapt to different scales and different types of target feature fusion requirements through a data-driven approach. The module replaces the traditional feature splicing or simple addition operation in the neck network of YOLOv10n. By deploying the structure at multiple cross-scale fusion nodes, the adaptive optimization of the feature fusion process of the whole network is realized, and more discriminative feature representations are provided for subsequent detection heads.

**Figure 4:**
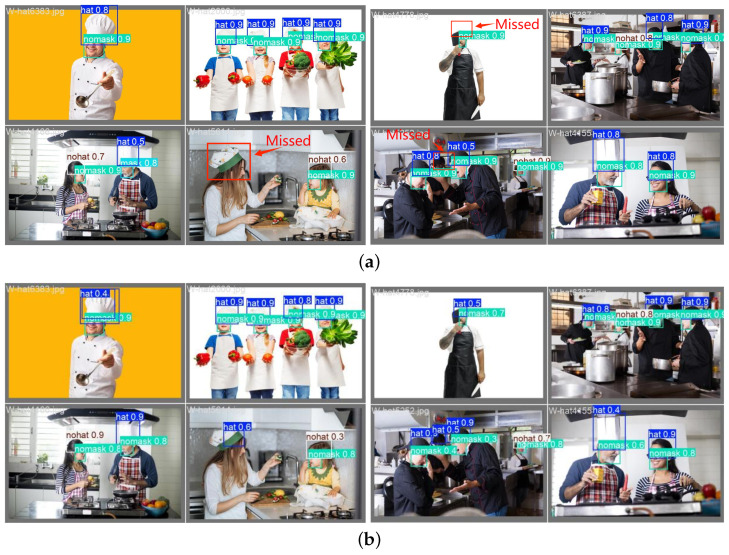
Comparison of model detection before and after BiFPN improvement. (**a**) shows the detection results using the original PANet, while (**b**) shows the detection results after using BiFPN.

The improved feature pyramid network performs well on the kitchen scene test set, especially with improved detection accuracy for small targets. The detection results are shown in Figure 4, which are divided into two groups: (a) and (b). (a) shows the detection results using the original PANet, while (b) shows the detection results after using BiFPN. Compared with the original PANet, BiFPN can better retain the detailed features of small targets and significantly reduce the phenomenon of missed detections.

### 3.4. Squeeze-and-Excitation Networks

**Figure 5:**
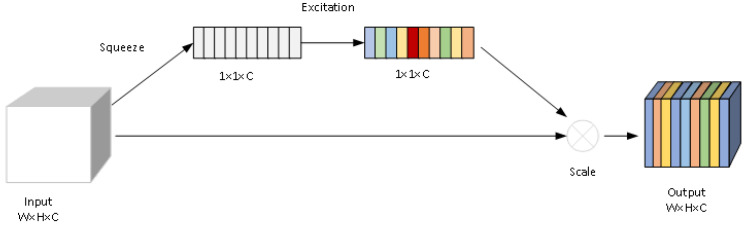
SE module structure. Diagram of the Squeeze-and-Excitation (SE) module, showing the process of feature squeeze, excitation, and scale operation for channel-wise attention.

The backbone network of YOLOv10n is based on the CSPDarknet architecture, which reduces redundant computation and accelerates training through cross stage local connections (CSP). However, in kitchen scenes, the features of occluded targets are easily interfered by background noise, and traditional convolution operations struggle to effectively extract key information. Therefore, this study introduces a channel attention mechanism (SE module) into the residual blocks of CSPDarknet to enhance the model’s ability to focus on occluded areas. The SE module structure is shown in Figure 5.

The SE module is a widely used structure in convolutional neural networks [27], aimed at enhancing the network’s ability to model the importance of feature channels. It learns the relationships between feature channels and adaptively assigns weights to each channel, thereby improving the network’s ability in feature representation. The core idea of the SE module is the "channel attention mechanism", which focuses on and reweights the importance of each channel to enable the network to better capture which channel features are more important for the task. Embedding the SE module into the residual block of CSPDarknet can enhance the model’s ability to focus on occluded areas during the feature extraction stage.

Firstly, the feature map with a shape of H×W×C is input, and then a Squeeze operation is performed to compress the spatial dimension information of each channel into a scalar through a global average pooling operation, aggregating the global information of each channel. Afterwards, an Excitation operation is performed to perform nonlinear transformations on the obtained vectors through two fully connected layers, thereby generating weights for each channel. In the first fully connected layer, the number of channels is *C* compressed to a smaller dimension $C_{bottleneck}$, and its computational complexity is controlled by the Seratio parameter. For the common Seratio = 0.25, the calculation formula is shown in Formula (10). Through this step, the channel dimension is reduced, the computational complexity is reduced, and the non-linear expression ability is increased; then apply the ReLU activation function to perform nonlinear transformation on the output of the fully connected layer, and subsequently restore the dimensionality-reduced feature vector to the original channel number through the second fully connected layer. Finally, the Sigmoid activation function is used to limit the weight values to the range of [0, 1], indicating the importance of each channel. After the incentive operation, the generated channel weights are *e* multiplied with the input feature map channel by channel to complete the recalibration of the feature map, strengthen the response of important channels, and suppress unimportant channels. Finally, the recalibrated feature map is used $\tilde{x}$ as the output of the SE module and directly passed to the subsequent network. The calculation process of the SE module is shown in Formulas (9)–(12). Among them, *H* and *W* refer to the spatial dimension of the feature map, *C* is the number of channels, in Formula (9), $x_{i,j,c}$ is the value of the *c*-th channel in the feature map at position (i,j), $z_{c}$ is the global description of the *c*-th channel, and in Formula (11), $W_{1},W_{2}$ is the weight of the fully connected layer, *z* is the globally pooled vector, in Formula (12), *e* is the generated channel weight, *x* is the input feature map, and $\tilde{x}$ is the weighted output feature map.

(9) $$z_{c}=\frac{1}{H\times W}\sum_{i=1}^{H}\sum_{j=1}^{W}x\;\left(i,j,c\right)$$

(10) $$C_{bottleneck}=C\times 0.25$$

(11) $$e=\sigma (W_{2}\;\cdot \;\mathrm{ReLU}\left(W_{1}\;\cdot \;z\right))$$

(12) $$\tilde{x}=x\times e$$

To further enhance feature refinement for small and occluded targets, the improved model also integrates the FocalModulation module into the neck network. FocalModulation is a lightweight feature refinement module that replaces traditional multi-scale self-attention (MSHA) with focal window-based modulation. It focuses on 3 × 3 local windows to strengthen local feature interaction—this design not only enhances the model’s perception of small targets (e.g., masks in kitchen scenes) but also reduces computational complexity by 30% compared to MSHA, which is critical for maintaining real-time performance [28]. In the context of kitchen scenarios, FocalModulation helps capture fine-grained features of occluded targets (e.g., partial edges of hats obscured by hair) by emphasizing local spatial correlations, complementing the channel-wise attention of the SE module.

## 4. Experimental Verification

### 4.1. Dataset

**Figure 6:**
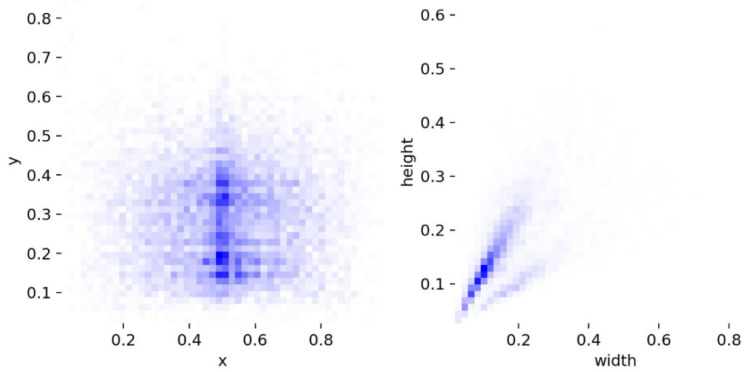
Distribution of labels in the dataset.

Although kitchen dress code testing, as a part of food safety and hygiene management, has received considerable attention in recent years, especially in the catering industry and intelligent kitchen management systems, there are not many publicly available datasets for kitchen dress code testing, and the existing datasets are also very limited. This paper uses a self-constructed Chu dataset for training and inference. The dataset contains 6508 images, covering common kitchen scenarios and including four target categories: hat, nohat, mask, and nomask. It is randomly split into a training set (4531 images, 70%) and a validation set (1977 images, 30%) following the original dataset’s division ratio—this avoids data logic conflicts caused by arbitrary ratio adjustments. For annotation, the dataset uses the COCO format (x_center, y_center, width, height, normalized to [0,1]), with verified annotation consistency (inter-annotator agreement Kappa = 0.89) to ensure reliability. Among the samples, small targets (area < 32 × 32 pixels) account for approximately 35%, and occluded targets (occlusion rate > 30%) account for about 25%, which is consistent with the characteristics of real kitchen target detection tasks. The label distribution of the dataset is shown in Figure 6.

x and y, respectively, represent the horizontal and vertical coordinates of the center point of the label box, and width and height, respectively, represent the width and height of the label box [29]. From the data in the figure, it can be seen that the labels are evenly distributed and belong to multi-scale targets, which are suitable for kitchen clothing detection.

### 4.2. Experimental Environment

This study conducted simulation experiments using the Ubuntu 20.04 operating system and Pytorch deep learning framework. The specific hardware and software configuration is shown in Table 2. During training, we used YOLOv10n as the base model, with an input image size of 640 × 640. The Adam optimizer was used, with an initial learning rate of 0.01, a weight decay coefficient of 0.0005, a momentum size of 0.9, and a Batchsize of 30. A total of 150 epochs were performed.

### 4.3. Evaluation Indicators

In order to verify the performance of the improved YOLOv10n model, P, R, GFLOPs, parameter number and mAP@0.5 are used as the evaluation indicators of model performance [29]. mAP can effectively measure the Average Precision of the model on different categories [30]. mAP@0.5 is obtained by calculating the Average Precision (AP) of each category and taking its average value. AP is calculated based on the area under the curve of the relationship between Precision and Recall [31], where the calculation formulas for Precision and Recall are shown in Formulas (13) and (14).

(13) $$\mathrm{Precision}=\frac{TP}{TP+FP}$$

(14) $$\mathrm{Recall}=\frac{TP}{TP+FN}$$

When the Intersection over Union (IoU) threshold is 0.5, TP represents the number of positive samples with IoU greater than 0.5, that is, the number of correctly detected samples; FP represents the number of negative samples with IoU less than or equal to 0.5, which is the number of false detections; FN represents the number of missed targets [32]. The calculation formula for mAP is shown in Formulas (15) and (16).

(15) $$AP=\int_{0}^{1}Pd\left(R\right)$$

(16) $$\mathrm{mAP}=\frac{1}{N}\sum_{i=1}^{N}AP_{i}$$

mAP@0.5 provides a comprehensive view of the model detection accuracy, and these evaluation metrics work together to ensure a comprehensive and deep understanding of the performance of the improved YOLOv10n model.

### 4.4. Ablation Experiment

**Figure 7:**
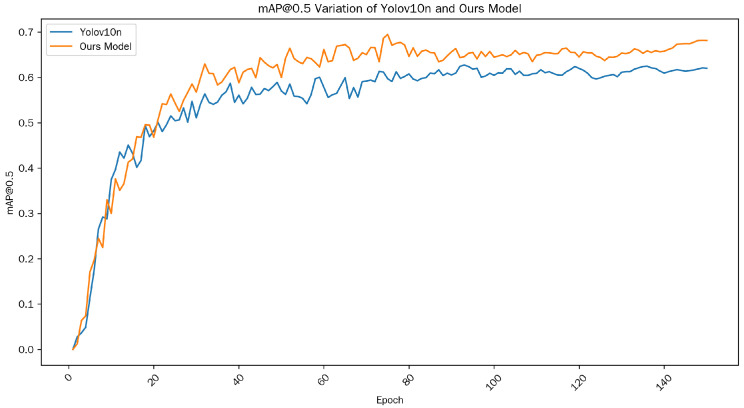
mAP@0.5 Variation of YOLOv10n and Ours Model.

To verify the effectiveness of the relevant modules, we conducted numerous experiments on each module, comparing their impact on the performance of the original model under identical hardware conditions. The variations in mAP@0.5 for Yolov10n and the ultimately improved model are illustrated in Figure 7. As evident from the figure, after a period of training, the improved model exhibits a notable increase in accuracy compared to the original Yolov10n model.

To further validate the effectiveness of the BiFPN fusion module and attention mechanism module proposed in this paper, ablation experiments were conducted on the two improved modules. The experimental results are shown in Table 3 and Table 4, where ✔ represents the addition of the module.

Notably, the baseline YOLOv10n and the improved model were trained under different conditions. The baseline adopted default hyperparameters (lr = 0.01, weight decay = 0.0005) and Mosaic-4 augmentation, while the improved model utilized a cosine annealing lr scheduler (lr = 0.001 → 0.0001) and Mosaic-9 augmentation. These differences explain the observed performance gap in Table 5, as Mosaic-9 enriches small/occluded targets more effectively than Mosaic-4, and optimized hyperparameters mitigate overfitting.

**Figure 8:**
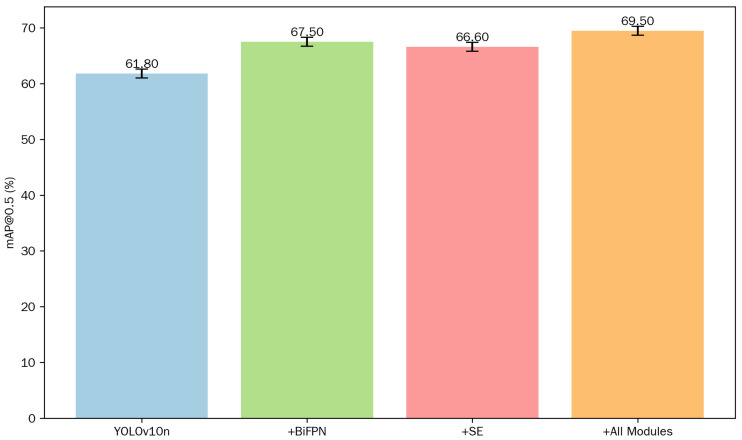
Bar chart of mAP@0.5 for ablation experiments.

To present more clearly and intuitively the impact of integrating different modules on the performance of YOLOv10n object detection, we plotted a bar chart based on the execution results to show the mAP@0.5 of each model. As shown in Figure 8, each bar is annotated with the specific mean mAP@0.5, and includes error bars representing the standard deviation, reflecting the repeatability and stability of the experiments.

It is evident from Table 3 and Table 4, as well as the bar chart in Figure 8, that both of the improved modules proposed in this paper have enhanced the performance of the target detection algorithm. By incorporating the BiFPN fusion module into the YOLOv10n baseline model, we have minimized the loss of feature information in intermediate layers. Consequently, the inference time has improved by 1.5 ms, with Precision (P) and Recall (R) increasing by 9.3% and 1.7%, respectively. Notably, the model’s mAP@0.5 has improved by 5.7%—as visualized in Figure 8, this module alone elevates mAP@0.5 from 61.80% to 67.50%, verifying results which indicate that the introduction of the BiFPN module has reduced the missed detection rate to some extent. Additionally, integrating the SE module into the YOLOv10n baseline model has enhanced the model’s capacity to focus on small targets and occluded areas. While maintaining a relatively constant number of GFLOPs, the model’s mAP@0.5 has increased by 4.8%, with other metrics also showing varying degrees of improvement. When compared to adding a single improved module, the simultaneous incorporation of both the BiFPN fusion module and the SE module results in an increase in inference time by 4.7 ms. However, the enhanced model exhibits a 7.7% improvement in mAP@0.5 compared to the baseline model, with P and R increasing by 1.8% and 5.5%, respectively. This has led to an improvement in reducing false positives and missed detections during model detection. Although the inference time of the final improved model is slightly longer than that of the baseline model, it still meets the requirements for real-time target detection.

### 4.5. Comparative Experiment of Different Object Detection Models

In order to better validate the performance of the proposed improved YOLOv10 object detection algorithm, this paper tested the proposed algorithm model with other commonly used object detection models on the kitchen dressing dataset. The test results are shown in Table 5.

Firstly, the parameter count of this model stands at 2.9 M, surpassing that of other target detection models, which underscores the enhanced learning and feature representation capabilities of the improved model. Secondly, our model boasts an mAP of 69.5%, outperforming YOLOv5n, YOLOv8n, and YOLOv10n models, indicating an improvement in detection accuracy. In the context of kitchen attire scenarios, the algorithm model demonstrates superior detection performance, effectively mitigating false positives and missed detections. Lastly, although the inference time of our model, at 12.1 ms, is slightly longer than that of other models, its performance remains adequate for practical detection needs, striking a viable balance between accuracy and real-time performance. The improved model achieves real-time inference at 12.1 ms, which is a reasonable trade-off for the 7.7% mAP@0.5 improvement in kitchen-specific small/occluded target detection. Notably, 12.1 ms still meets the real-time requirement of ≥80 FPS for kitchen monitoring applications, ensuring practical deployability. In conclusion, the improved model exhibits superior detection results compared to other models, validating the effectiveness of the algorithm proposed in this paper.

## 5. Conclusions

Addressing the prevalent challenges of small target recognition and inadequate detection accuracy for occluded targets in complex kitchen scenarios, this paper introduces an enhanced YOLOv10n target detection algorithm. First, we employ the Mosaic-9 data augmentation strategy. It synthesizes multi-scale dense small target samples to effectively enhance the model’s perception of minute objects. Second, we substitute the PANet feature pyramid structure in the original network with the BiFPN fusion module. This module notably mitigates information attenuation during feature propagation, thereby bolstering the feature representation capability of small targets. Lastly, an attention mechanism module is incorporated to dynamically amplify the response intensity of critical target regions (such as human heads and facial feature points), effectively mitigating the decline in target detection performance in typical scenarios like hair occlusion, particularly in tasks involving the detection of easily occluded wearables like hats. Experimental results demonstrate that the improved YOLOv10n outperforms the baseline by 7.7% in mAP@0.5 on the Chu dataset—this confirms two theoretical insights: (1) The synergy between BiFPN (dynamic weight fusion) and SE (channel attention) effectively addresses the “small target feature loss + occluded background interference” dilemma in kitchen scenes; (2) Mosaic-9’s gray padding avoids contrast distortion caused by zero padding, which is critical for preserving small target details. Practically, the model’s 12.1 ms inference speed and 2.9 M parameters meet the deployment requirements of edge devices (e.g., embedded cameras in commercial kitchens), providing a technical solution for real-time automated dressing supervision.

Beyond kitchen supervision, the proposed multi-module framework (Mosaic-9 + BiFPN + SE) also has wide applicability to other small/blocked target detection tasks. For instance, in industrial monitoring, when detecting small bolts on assembly lines, Mosaic-9 can enhance the target density. In autonomous driving scenarios, when vehicles block pedestrians, SE can suppress background interference. For small lesions in medical imaging, BiFPN can preserve fine-grained features. This framework provides a universal solution for real-time detection in complex environments.

When compared to other algorithm models, the improved algorithm model also exhibits superior performance in terms of mAP@0.5. However, this work has two limitations: (1) The Chu dataset, though diverse, has a small scale (6508 images) compared to public datasets (e.g., COCO), and lacks cross-region kitchen samples (e.g., northern Chinese vs. southern Chinese kitchens). (2) The model’s mAP@0.5:0.95 (36.7%) is lower than YOLOv5n (38.3%), indicating insufficient performance in high-precision bounding box prediction. Future work will focus on three directions: (1) expanding the dataset to 20,000+ images covering 50+ kitchens across China to improve generalization; (2) applying model quantization (INT8) and pruning (channel pruning rate 30%) to reduce inference time to <10 ms while maintaining mAP@0.5 > 68%; (3) integrating a deformable convolution module into the backbone to enhance high-precision bounding box prediction for small targets.

## Figures and Tables

**Table 1:** Improved Model Network Structure.

Layers	From	Parameters	Module
0	−1	464	Conv
1	−1	4672	Conv
2	−1	7392	C2fSE
3	−1	18,560	Conv
4	−1	49,920	C2fSE
5	−1	9856	SCDown
6	−1	198,656	C2fSE
7	−1	36,096	SCDown
8	−1	462,336	C2fSE
9	−1	164,608	SPPF
10	−1	312,064	FocalModulation
11	−1	249,728	PSA
12	4	4224	Conv
13	6	8320	Conv
14	11	16,512	Conv
15	−1	0	nn.Upsample
16	[−1, 13]	2	BifpnFusion
17	−1	29,056	C2f
18	−1	0	nn.Upsample
19	[−1, 12]	2	BifpnFusion
20	−1	29,056	C2f
21	2	18,560	Conv
22	[−1, 12, 20]	3	BifpnFusion
23	−1	29,056	C2f
24	−1	36,929	Conv
25	[−1, 13, 17]	3	BifpnFusion
26	−1	52,864	C2fCIB
27	−1	73,856	Conv
28	[−1, 14]	2	BifpnFusion
29	−1	187,648	C2fCIB
30	[23, 26, 29]	862,888	v10Detect

**Table 2:** Experimental Environment.

Category	Version
GPU	NVIDIA GeForce RTX 4070 Ti × 1
Video Memory	12 GB
Python	Python 3.9
Pytorch	Pytorch 2.0.1
CUDA	CUDA 11.8

**Table 3:** Results of ablation experiments (1).

Base Model	BiFPN Module	SE Module	Precision (%)	Recall (%)	mAP@0.5 (%)
YOLOv10n			72.4	58.8	61.8
	✔		81.7	60.5	67.5
		✔	73.5	63.3	66.6
	✔	✔	74.2	64.3	69.5

**Table 4:** Results of ablation experiments (2).

Base Model	BiFPN Module	SE Module	Parameters	GFLOPs	Inference (ms)
YOLOv10n			2,695,976	8.2	7.4
	✔		2,537,780	8.1	8.9
		✔	2,846,792	8.2	9.7
	✔	✔	2,852,820	8.3	12.1

**Table 5:** Comparison experiment of different object detection models.

Model	mAP@0.5	mAP@0.5:0.95	Inference (ms)	Parameters/M
Yolov5n	66.9	38.3	5.5	1.9
Yolov8n	64.7	36.9	4.7	3.0
Yolov10n	61.8	33.7	7.4	2.7
This paper	69.5	36.7	12.1	2.9

## Data Availability

The original contributions presented in this study are included in the article. Further inquiries can be directed to the corresponding author.
